# Evidence of zoonotic leprosy in Pará, Brazilian Amazon, and risks associated with human contact or consumption of armadillos

**DOI:** 10.1371/journal.pntd.0006532

**Published:** 2018-06-28

**Authors:** Moises B. da Silva, Juliana M. Portela, Wei Li, Mary Jackson, Mercedes Gonzalez-Juarrero, Andrea Sánchez Hidalgo, John T. Belisle, Raquel C. Bouth, Angélica R. Gobbo, Josafá G. Barreto, Antonio H. H. Minervino, Stewart T. Cole, Charlotte Avanzi, Philippe Busso, Marco A. C. Frade, Annemieke Geluk, Claudio G. Salgado, John S. Spencer

**Affiliations:** 1 Laboratório de Dermato-Imunologia, Instituto de Ciências Biológicas, Universidade Federal do Pará, Marituba, Pará, Brazil; 2 Universidade Federal do Oeste do Pará, Santarém, Pará, Brazil; 3 Department of Microbiology, Immunology, and Pathology, Mycobacteria Research Laboratories, Colorado State University, Fort Collins, Colorado, United States of America; 4 Unidade de Referência Especializada em Dermatologia Sanitária do Estado do Pará - URE Dr. Marcelo Candia, Marituba, Pará, Brazil; 5 Spatial Epidemiology Laboratory, Universidade Federal do Pará, Campus Castanhal, Pará, Brazil; 6 École Polytechnique Fédérale de Lausanne, Lausanne, Switzerland; 7 Dermatology Division of the Department of Internal Medicine, Faculdade de Medicina de Ribeirão Preto da Universidade de São Paulo, Ribeirão Preto, São Paulo, Brazil; 8 Department of Infectious Diseases, Leiden University Medical Center, Leiden, The Netherlands; Swiss Tropical and Public Health Institute, SWITZERLAND

## Abstract

*Mycobacterium leprae* (*M*. *leprae*) is a human pathogen and the causative agent for leprosy, a chronic disease characterized by lesions of the skin and peripheral nerve damage. Zoonotic transmission of *M*. *leprae* to humans by nine-banded armadillos (*Dasypus novemcinctus*) has been shown to occur in the southern United States, mainly in Texas, Louisiana, and Florida. Nine-banded armadillos are also common in South America, and residents living in some areas in Brazil hunt and kill armadillos as a dietary source of protein. This study examines the extent of *M*. *leprae* infection in wild armadillos and whether these New World mammals may be a natural reservoir for leprosy transmission in Brazil, similar to the situation in the southern states of the U.S. The presence of the *M*. *leprae*-specific repetitive sequence RLEP was detected by PCR amplification in purified DNA extracted from armadillo spleen and liver tissue samples. A positive RLEP signal was confirmed in 62% of the armadillos (10/16), indicating high rates of infection with *M*. *leprae*. Immunohistochemistry of sections of infected armadillo spleens revealed mycobacterial DNA and cell wall constituents *in situ* detected by SYBR Gold and auramine/rhodamine staining techniques, respectively. The *M*. *leprae*-specific antigen, phenolic glycolipid I (PGL-I) was detected in spleen sections using a rabbit polyclonal antibody specific for PGL-I. Anti-PGL-I titers were assessed by ELISA in sera from 146 inhabitants of Belterra, a hyperendemic city located in western Pará state in Brazil. A positive anti-PGL-I titer is a known biomarker for *M*. *leprae* infection in both humans and armadillos. Individuals who consumed armadillo meat most frequently (more than once per month) showed a significantly higher anti-PGL-I titer than those who did not eat or ate less frequently than once per month. Armadillos infected with *M*. *leprae* represent a potential environmental reservoir. Consequently, people who hunt, kill, or process or eat armadillo meat are at a higher risk for infection with *M*. *leprae* from these animals.

## Author summary

Armadillos have been shown to be a natural reservoir of *Mycobacterium leprae* infection in the southern states of the U.S. and have been implicated in the zoonotic transmission of leprosy to humans. To investigate this in Brazil, we conducted surveys of armadillos in western Pará state in the Brazilian Amazon region where leprosy is hyperendemic in humans. Individuals living in the small town of Belterra were surveyed for the extent and frequency of interaction with armadillos (hunting, preparing the meat for cooking, or eating the meat for food). We also took samples of liver and spleen from armadillos to look for *M*. *leprae* infection in the tissues. We found that a majority of residents had some contact with armadillos (~65%) and that infection by *M*. *leprae* in armadillos in this area was also very high (62%). Those individuals who ate armadillo meat more than once a month had a significantly higher antibody titer to the *M*. *leprae*-specific antigen, PGL-I. Understanding the dynamics of leprosy transmission in different geographic regions and knowing the behavioral risks of humans interacting with potentially infected animals will help clarify the relative risk of zoonotic transmission of leprosy in this region.

## Introduction

The human pathogen, *M*. *leprae*, causes leprosy, a slowly developing chronic granulomatous disease mainly affecting the skin and peripheral nerves, resulting in disfiguring lesions and progressive nerve damage that can lead to muscle weakness or atrophy, bone loss, amputations and blindness [1]. The discovery of the bacillus was credited by work by the Norwegian physician Gerhardt Henrik Armauer Hansen in 1873. The name Hansen’s disease (hanseníase in Portuguese) is used in Brazil to lessen the stigma associated with the common name. Multidrug therapy (MDT) was introduced by the World Health Organization (WHO) in the mid-1980’s, and has been provided free of charge upon diagnosis worldwide for over 30 years. The worldwide prevalence of the disease has decreased from >5 million cases in the 1980’s to <200,000 by 2016. Nevertheless, the WHO recorded 214,783 new cases in 2016 [2], slightly higher than the previous year, with around 80% of all cases being found in only three countries: India, Brazil and Indonesia. The Americas recorded 27,356 cases in 2016, with Brazil having 25,218, or 92.2% of the total. Brazil is still the only country in the world that has not reached the WHO goal of <1 new case per 10,000 population and is currently at around 1.2/10,000 nationally (SINAN, Brazil’s Notifiable Diseases Information System) [3]. However, there is a wide variation in new case detection in regional areas in Brazil, ranging from as low as <0.2/10,000 in the southern state of Rio Grande do Sul to >4/10,000, considered hyperendemic, in the central (Mato Grosso, Rondônia), north (Pará, Tocantins), and northeastern states (Maranhão) [4]. Historically, Pará and the Amazon region have recorded some of the highest new case detection rates in the country, despite having one of the lowest population densities [5]. Reasons behind this have been well-documented, and include living in a hyperendemic area, low human development index (HDI, which combines life expectancy at birth, per capita income, and education level), living with an untreated index case or within 200 meters of a case, high household density (>2 people per bedroom), poor nutritional status, and lack of healthcare availability [6,7].

The disease causes a broad array of skin lesions, nerve damage, peripheral neuropathy and anesthesia. The variability in clinical manifestations of leprosy is aligned with the hosts’ abilities to mount effective immune responses to *M*. *leprae*, dependent on the interplay of both cell mediated and humoral responses [8]. There is an overall genetic resistance towards developing leprosy, with over 90% of people having a natural immunity [9]. For those individuals who do progress to disease, the interplay of cell mediated and humoral immunity to *M*. *leprae* becomes clear from the well-known immunological and clinical leprosy spectrum, ranging from tuberculoid (TT/BT) or paucibacillary (PB) leprosy to lepromatous (BB/BL/LL) or multibacillary (MB) leprosy, defined by Ridley-Jopling classification based on histopathology and bacillary load [10]. PB patients generally show high cellular responses to *M*. *leprae* antigens *in vitro* as measured by the production of Th1 cytokines, particularly IFN-γ, and have low antibody titers to *M*. *leprae*-specific antigens. MB patients have lost some or all capacity to mount a cell mediated response due to T cell anergy but have high antibody titers to *M*. *leprae* antigens, particularly to the *M*. *leprae*-specific glycolipid, PGL-I [11,12]. The humoral response to PGL-I in leprosy patients, mainly IgM, correlates very well with the BI (bacillary index) and is highly specific, and the anti-PGL-I titer can be easily assessed using either the standard ELISA assay or in a lateral flow device [13–16] using the synthetic di- or trisaccharide of PGL-I linked to a protein carrier, bovine or human serum albumin (BSA or HSA) derivatives. Anti-PGL-I IgM seropositivity has been used to reliably assess the prevalence of leprosy in endemic areas, since a positive titer is a definitive biomarker of prior *M*. *leprae* infection [17–21].

Since *M*. *leprae* cannot be grown *in vitro* on axenic medium, the mechanism of human transmission has long been debated. The most widely accepted theory is that untreated index cases, particularly MB individuals who are capable of discharging an estimated 10^7^ bacilli per day from nasal secretions [22], are the main source of transmission via the aerosol route. A number of studies have shown that consanguineous individuals of index cases living for extended periods, months or years, in the same household (household contacts, HC) have the highest risk for developing disease [23–26].

Besides human contact, the only other known transmission route is from human contact with armadillos that have been naturally infected with *M*. *leprae*. Armadillos have an immune system that responds similarly to *M*. *leprae* infection, and essentially recapitulates the spectrum of human disease [27]. This includes developing progressive nerve damage and characteristic ulcers and skin lesions due to loss of sensation in the feet and face and even develop high antibody titers to PGL-I and other *M*. *leprae* proteins [28]. As early as 1975, wild armadillos were found to be naturally infected with *M*. *leprae*, but it was later shown that sylvan leprosy had existed in this species for decades before they were artificially infected [29,30]. Surveys in Texas and Louisiana showed that disease prevalence rates among nine-banded armadillos were >20% in some areas [31].

In this study we investigated armadillos from an area in Brazil that is hyperendemic for leprosy in the town of Belterra in western Pará state to explore whether sylvan leprosy exists in wild armadillos in this area. We also performed a survey of the relationship of the people in this town with armadillos to determine if any activities related to hunting, killing, preparing or handling the armadillo meat for consumption, as well as the frequency of armadillo in the diet had any effect on the anti-PGL-I titer.

## Methods

### Ethics statement

The research protocol was approved by the institutional review boards at the Universidade Federal do Oeste do Pará (UFOPA) and the Universidade Federal do Pará (UFPA) (IRB protocol #517.394 ICS/UFPA) and Colorado State University (IRB protocol 15-6340H) and conducted in accordance with the guidelines of the Declaration of Helsinki. All individuals who agreed to participate read and signed a written informed consent document. Environmental approval of wild armadillo tissue sampling was obtained with ICMBio authorization for research activities (SISBIO 44831–1). Environmental approval of wild armadillo tissue sampling was obtained with ICMBio authorization for research activities (SISBIO 44831–1), Ministério do Meio Ambiente, Instituto Chico Mendes de Conservação da Biodiversidade—ICMBio, Sistema de Autorização e Informação em Biodiversidade—SISBIO, Brazil.

### Study location

The survey site chosen was the city of Belterra in western Pará state where two rural communities exist at 92 Km (São Jorge) and 135 Km (Corpus Christi) on the Santarém-Cuiabá highway, located roughly by coordinates at 2° 38'S and 54° 56'W. The city has a total area of 4,398 km^2^ with 17,036 inhabitants (IBGE, Instituto Brasileiro de Geografia e Estatistica, 2015) and is roughly 1,300 Km southwest from the capital city of Belém. The city was chosen because of its proximity to Santarém where one of the affiliated universities is located (UFOPA) and because it was determined that there was a high density of armadillos living in the surrounding forest with a high percentage of people living in this rural area that hunted or consumed armadillos as food.

### Human subject population

A total of 146 individuals living in the town of Belterra were asked to participate in a research protocol approved by the institutional review boards at the Universidade Federal do Oeste do Pará (UFOPA) and the Universidade Federal do Pará (UFPA) (IRB protocol #517.394 ICS/UFPA) and Colorado State University (IRB protocol 15-6340H) and conducted in accordance with the guidelines of the Declaration of Helsinki. All individuals who agreed to participate read and signed a written informed consent document. In the case of minors, consent was obtained from a parent or guardian of the child. All individuals received a free dermatologic exam performed by experienced leprosy clinicians, and a sample of blood was drawn from each person by a trained phlebotomist for anti-PGL-I titer assessment. Besides demographic information for each individual, a survey included questions about the extent of contact with armadillos (hunting armadillos in the forests; killing and/or handling the armadillo meat for consumption; and the frequency of eating armadillo meat). The diagnosis of leprosy was performed using internationally accepted clinical criteria based on the presence of skin lesions with sensory loss and/or nerve damage associated with nerve swelling and pain, muscle weakness or disability. Individuals diagnosed with leprosy received free MDT treatment from their local basic health unit.

### Assessment of anti-PGL-I titer by ELISA

An indirect ELISA was used to measure the anti-PGL-I IgM titer of all of the serum samples tested at a 1:300 dilution using a protocol previously reported [32]. The cut-off for positivity was established at an optical density (O.D.) of 0.295 based on the average plus three times the standard deviation of healthy subjects from a hyperendemic area as reported. The O.D. for each well was read at 490 nm using an ELISA plate reader.

### Collection of armadillo liver and spleen samples for detection of RLEP sequence by PCR

By collaborating with local residents who hunted armadillos in the surrounding forest in the area, we obtained samples of armadillo liver and spleen from freshly killed animals from different households from residents in both villages in Belterra (“A” armadillos from Corpus Christi, n = 3, and “B” armadillos from São Jorge, n = 13). A sterile scalpel blade was used to excise several pieces of tissue, at least 1cm^3^ each, and placed in individual sterile 5 ml plastic tubes containing 70% ethanol to fix the specimen before DNA extraction. A fresh scalpel blade was used for each tissue for each animal. Environmental approval of wild armadillo tissue sampling was obtained with ICMBio authorization for research activities (SISBIO 44831–1). DNA was extracted in the laboratory from approximately 1 gm of fixed tissue using the Qiagen DNeasy Blood and Tissue Kit (Qiagen, Germantown, MD) following the protocol supplied by the manufacturer. The amount of DNA in each sample was quantified using nanodrop. The *M*. *leprae-*specific repetitive sequence, RLEP, was amplified by PCR using a Qiagen Multiplex PCR Kit (Qiagen) and primers (LP1 forward primer:

5'-TGCATGTCATGGCCTTGAGG-3' and LP2 reverse primer:

5'-CACCGATACCAGCGGCAGAA-3') that amplifies a 129-base pair fragment found in the *M*. *leprae* genome. The primer sequences and the protocol used were adapted from Donoghue [33] using the following PCR protocol: denaturation at 95°C for 15 min, 40 cycles of denaturation at 94°C for 30 s, primer annealing at 58°C for 40 s, extension at 72°C for 30 s, and final extension at 72°C for 10 min. Each reaction set included a positive control tube using purified *M*. *leprae* DNA (2 ng) and a negative control tube without template DNA.

### Staining of *M*. *leprae* in armadillo spleen sections to detect mycobacterial DNA, cell wall constituents and *M*. *leprae* PGL-I

At the time of necropsies, samples of spleen were aseptically removed and prepared for histological examination and specific staining procedures for visualization of mycobacteria. Tissue samples were fixed in 4% paraformaldehyde in phosphate buffered saline (PBS), embedded in paraffin and sectioned to 5 μm thickness. Subsequent tissue sections were mounted on glass slides, deparaffinized and stained either with auramine-rhodamine (AR) or SYBR Gold fluorescent stain [34]. For AR staining, each section was stained using the TB Fluorescent Stain Kit T (Becton-Dickinson, Sparks, Maryland) per manufacturer's instructions. The AR stain was added to the deparaffinized section on the slide and incubated in the dark at room temperature for 25 min, washed in acid-alcohol (0.5% HCl in 70% isopropanol) for no more than 3 min, followed by washing with water and counterstaining with potassium permanganate (0.5%) for 4 min. The slides were washed again in water and then mounted with Prolong Gold anti-fade mounting medium (Invitrogen, Carlsbad, California).

SYBR Gold staining was performed using a dilution of SYBR Gold fluorescence dye at 1:1,000 in a stain solution of 0.85 M phenol in a 60% glycerol/14% isopropanol solution in distilled water. The slides were heated on a block at 65°C for 5 min and then cooled at room temperature for an additional 5 min. The tissue sections were washed with acid alcohol (0.5% HCl in 70% isopropanol) for 3 min, then washed with water and counterstained with hematoxylin QS (Vector Laboratories, Inc., Burlingame, CA), for 5–10 s. The excess hematoxylin was washed away with distilled H_2_O and slides were subsequently stained with 4,6-diamidino-2-phenylindole (DAPI; Sigma Chemical, St. Louis, MO) at 200 ng/ml final concentration for 10 min and washed again with water. Slides were mounted with Prolong Gold antifade mounting media. All stained sections were visualized using Zeiss 510 confocal microscopy and Zen software.

For PGL-I antigen localization, deparaffinized spleen sections were covered with peroxidazed 1 solution (Biocare Medical, Concord, CA) for 5 min to block endogenous peroxidase, followed by antigen retrieval procedure (Dako, Agilent Technologies, Carpinteria, CA). Background sniper (Biocare Medical) was used for 10 min to block nonspecific binding sites, followed by addition of a 1:500 dilution of rabbit polyclonal antisera specific for PGL-I (produced at Colorado State University) and incubated for 2 h in a humidified chamber at room temperature. Thereafter, the sections were washed 3 times for 5 min in PBS and incubated with a secondary goat anti-rabbit IgG-F(abʹ)_2_ coupled to horseradish peroxidase (Santa Cruz Biotechnology, Santa Cruz, CA) for 1 h. After washing the sections with PBS, the substrate (ImmPACT from AEC, Vector Laboratories) was added for 10 min or until brownish-red color was developed. Slides were washed with PBS and counterstained with hematoxylin, followed by visualization using light microscopy.

Control *M*. *leprae* infected and non-infected armadillo spleens that were formalin fixed and paraffin embedded were generously provided by Dr. Maria Pena from the National Hansen’s Disease Program (NHDP), Baton Rouge, LA. Sections of control tissues were stained for PGL-I antigen as above. Sections were also stained by the Fite Faraco modification of the Ziehl-Neelsen staining technique to identify acid fast bacilli in control and wild armadillo tissues.

### Statistical analysis

The anti-PGL-I titer expressed as the O.D. was compared with the epidemiologic data and individual’s contact with armadillos by the Mann-Whitney test. The frequency of anti-PGL-I positives in the total population and among diagnosed leprosy patients were estimated between variables by the ratio of crossed products-Odds Ratio (OR) and their 95% confidence intervals using the χ2 test or Fisher’s exact test to verify the significance of associations. The GraphPad Prism (GraphPad Software Inc., La Jolla, CA) statistical software program was used. The threshold for statistical significance was set at 5%.

## Results

### High infection rates in armadillos from Belterra

**Fig 1:**
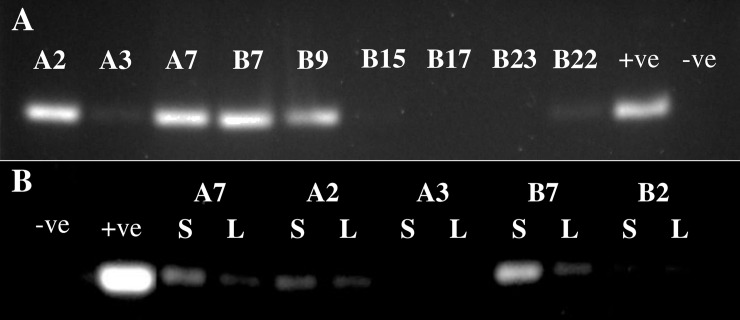
Detection of RLEP sequence by PCR in armadillo tissues. A) Analysis of PCR RLEP product from spleen samples from nine different armadillos. B) Analysis of RLEP from paired samples of liver (L) and spleen (S) from five different armadillos. The signal from positive samples is consistently stronger in the spleen for each individual. The positive control (+ve) reaction included purified *M*. *leprae* DNA, 2 ng, while the negative control (-ve) lacked DNA template.

Sixteen armadillos with an average weight of 3.7 Kg and 54.3 cm in length were captured by local residents by hunting in the surrounding tropical forest located around the communities. When extracted DNA from spleen tissues was tested for the presence of the RLEP repetitive sequence by PCR, 10/16 armadillos were positive (62%), indicating that a high percentage of these animals were infected with *M*. *leprae* (Fig 1A). Since *M*. *leprae* infects internal organs in armadillos, particularly spleen and liver, we examined both tissues to determine if infection was consistently found in these organs. Of five armadillos examined from the same group, armadillos that were RLEP positive in the spleen were also RLEP positive in the liver while animals that were RLEP negative were negative in both (Fig 1B). In addition, the signal strength in positive animals was much stronger in spleen than in liver. This finding is consistent with yields of *M*. *leprae* from experimentally infected armadillos from NHDP, with the yields in the spleen averaging 4 to 10 fold higher than in liver. The sequence of the RLEP product produced by PCR was confirmed to be identical to the published sequence found in *M*. *leprae* for all positive samples by submitting the PCR product for sequencing. Whole genome sequence results confirmed *M*. *leprae* sequence in three animals (A7, B21 and B22) with ~2X coverage.

**Fig 2:**
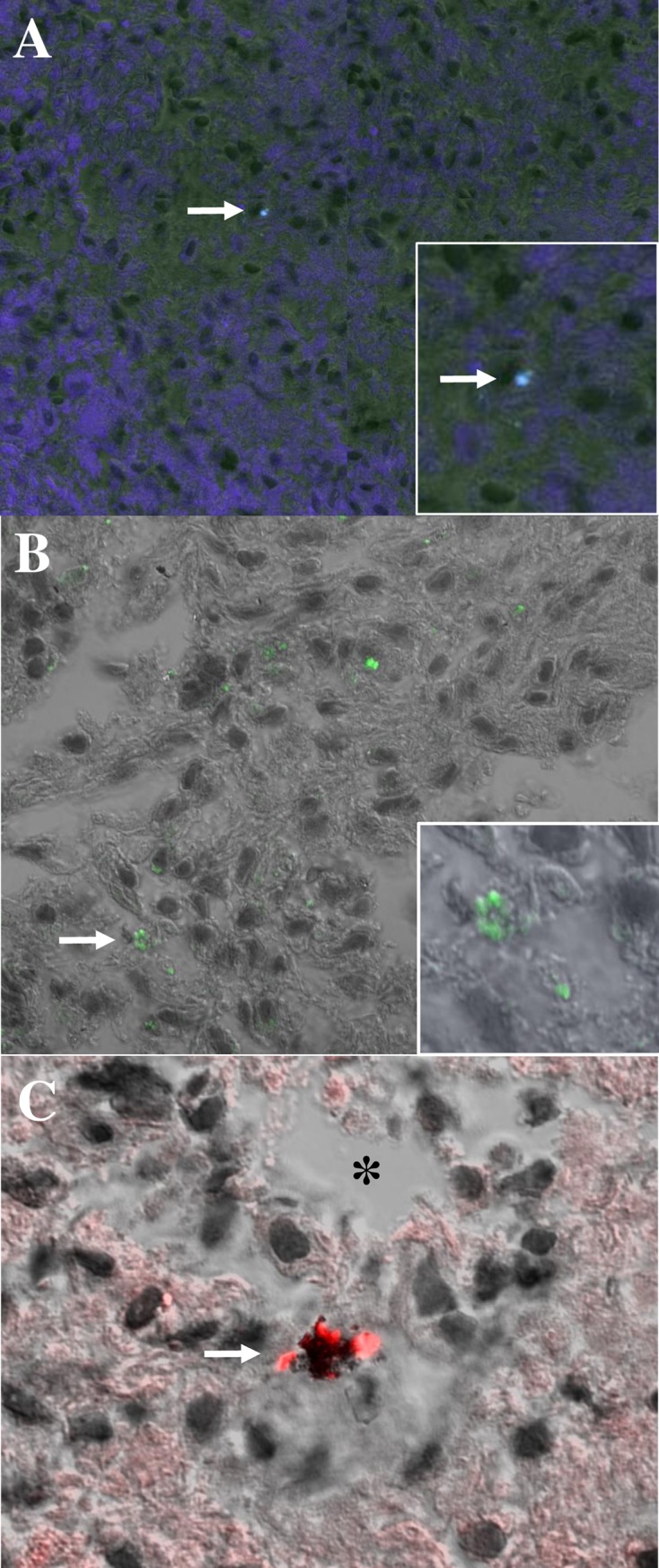
Staining of mycobacteria *in situ* in *M*. *leprae* infected armadillo spleen sections. A) SYBR Gold staining (blue) of *M*. *leprae* in armadillo spleen A7, arrow denotes stained bacillus. Insert, enlarged area showing stained bacillus. B) SYBR Gold staining (green) of *M*. *leprae* in armadillo spleen B7. Arrow denotes stained cluster of bacilli, enlarged in insert. C) Auramine/rhodamine stained cluster (red) of bacilli (arrow) located within an apoptotic cell next to a cell-free necrotic zone (*) in armadillo spleen A7.

**Fig 3:**
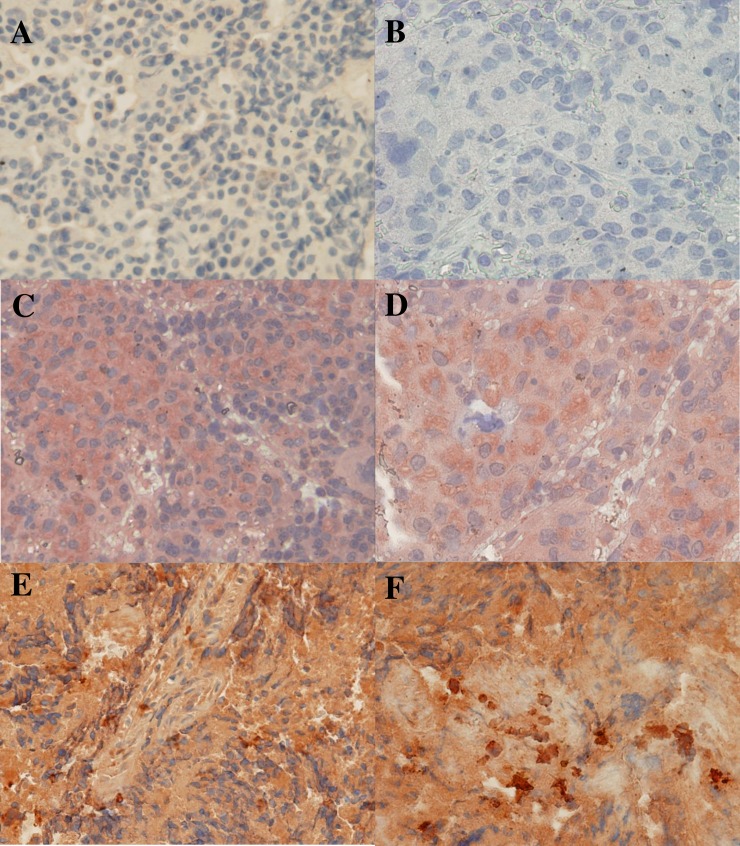
Immunohistochemical staining of armadillo spleen sections for the *M*. *leprae*-specific antigen, PGL-I, with a rabbit polyclonal antibody raised against this antigen. A) Wild infected armadillo B7 section stained with pre-immune rabbit serum, negative control; B) NHDP uninfected armadillo 13–02 stained with rabbit anti-PGL-I, negative control; C) NHDP infected armadillo 11K902 stained with anti-PGL-I showing diffuse brown staining, positive control; D) Higher magnification of NHDP infected armadillo 11K902 stained with anti-PGL-I, positive control; E) Wild infected armadillo A7 stained with anti-PGL-I, showing diffuse brown staining. F) Higher magnification of wild infected armadillo A7 stained with anti-PGL-I, showing diffuse brown staining with more intense clusters.

**Fig 4:**
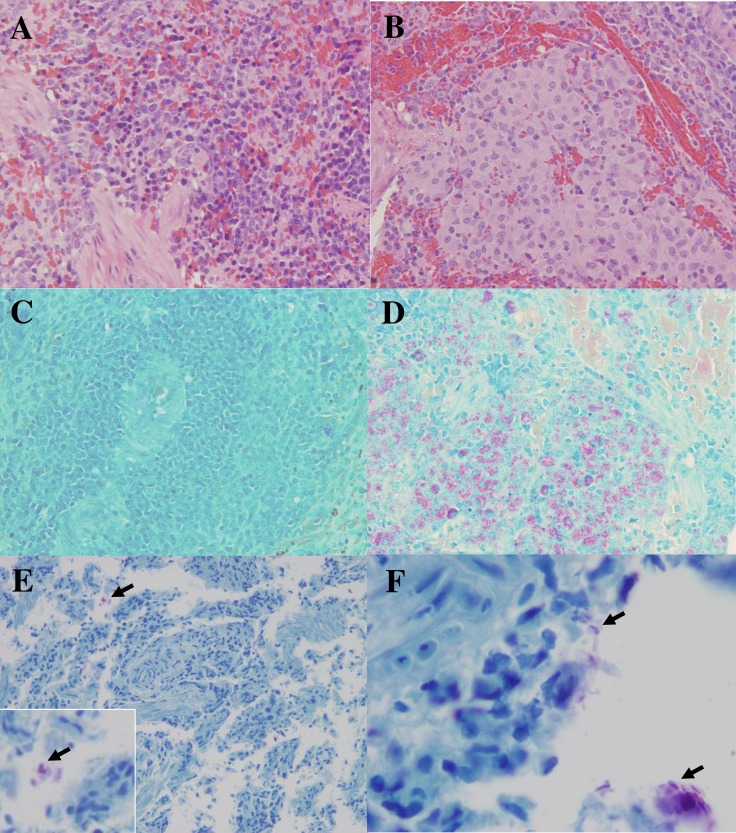
Hematoxylin and eosin (H&E) and Fite Faraco (acid fast) staining of non-infected and infected armadillo spleen sections. A) Non-infected control NHDP armadillo spleen (13–02) stained by H&E to show normal splenic architecture; B) *M*. *leprae* infected NHDP armadillo spleen (11I302) stained by H&E; C) Non-infected control NHDP armadillo spleen (12–70) stained by Fite Faraco method showing only counterstain; D) Many acid fast bacilli (red clusters) in *M*. *leprae* infected NHDP armadillo spleen (11I302) revealed by Fite Faraco stain; E) Wild armadillo (A2) spleen section stained with Fite Faraco, arrow pointing to acid fast bacilli at lower magnification and enlarged in insert; F) Wild armadillo (A2) spleen section stained with Fite Faraco revealing clusters of acid fast bacilli (arrows).

Thin sections of paraffin embedded armadillo spleens were stained to identify *M*. *leprae in situ* using SYBR Gold (Fig 2A and 2B) and auramine/rhodamine (Fig 2C), staining techniques which are highly specific for DNA or mycobacterial cell wall components, respectively. A rabbit polyclonal serum raised against the *M*. *leprae*-specific PGL-I antigen was used to stain spleen sections by immunohistochemistry. Wild or control infected armadillo sections stained with the pre-immune serum and control uninfected armadillo spleens from NHDP stained with the anti-PGL-I serum were negative (Fig 3A and 3B, respectively). Diffusely localized PGL-I antigen was seen in spleen sections from control infected NHDP armadillos (Fig 3C and 3D) and wild infected armadillos (Fig 3E and 3F). In all cases, positive staining was identified using these techniques only in infected armadillo tissues. Hematoxylin and Eosin (H&E) and Fite Faraco staining (acid fast staining) of control NHDP infected and noninfected armadillo and infected wild armadillo tissues sections were included to show the architecture of the spleen and acid fast bacilli (Fig 4A–4F).

### Characteristics of the individuals studied

**Fig 5:**
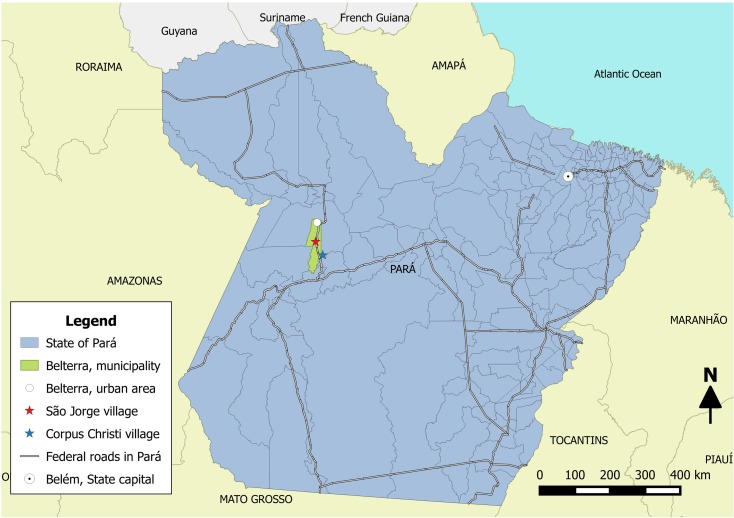
Map of study area. Municipality of Belterra in western Pará, and the villages of São Jorge at Km 92 and Corpus Christi at Km 135 on the Santarém-Cuiabá highway.

Of the 146 people surveyed in the town of Belterra, the subjects were divided equally (n = 73) between two villages located within the town 44 Km apart, namely the village of São Jorge, where residents of 32 households participated, and the village of Corpus Christi, where 31 households were surveyed (Fig 5). Four new cases were diagnosed based on clinical signs and symptoms during our visit (2.7%), and we identified 3 patients who had been previously diagnosed and received treatment, bringing the total to 7 patients identified. Testing for anti-PGL-I titer by ELISA showed that 92/146 (63%) were positive. These numbers are consistent with previously published data from our group on new case detection and anti-PGL-I positivity rates for individuals living in hyperendemic areas in the state of Pará.

The four newly diagnosed cases and three other individuals who had completed treatment displayed a wide range of characteristics (Table 1). The age range was from 13 to 72, with two residing in the São Jorge community, while five lived in Corpus Christi. Four of these individuals hunted armadillos resulting in a relatively high risk (OR 6.73, 95% CI 1.41–32.09, *p* = 0.02). Although hunting armadillos in the forest was exclusively a male activity and handling or cleaning the armadillo meat to prepare it for cooking was primarily a responsibility for women, 6 of these male patients indicated that they also participated in cleaning or preparing armadillo meat for cooking and also ate armadillo regularly, although these factors were not significant, probably due to the small sample size. This suggests that the majority of the leprosy patients had patterns of exposure (hunting, handling, and eating) to armadillos that were all at the high end relative to other groups in the village.

**Table 1:** Risk factors associated with leprosy patients living in rural communities in the municipality of Belterra, Pará.

Risk factors	Leprosy* Patient/Total (%)	OR	95% CI	p**
**Village**				
São Jorge	2/73 (2.7%)	0.38	0.07–2.04	0.44
Corpus Christi	5/73 (6.9%)	2.60	0.49–13.92	
**Previous contact with a leprosy patient**				
Yes	3/37 (8.1%)	2.31	0.49–10.87	0.37
No	4/109 (3.7%)			
**Gender**				
Male	5/63 (7.9%)	3.49	0.65–18.63	0.12
Female	2/83 (2.4%)	0.29	0.05–1.53	
**Age group**				
≤20	1/34 (2.9%)	0.54	0.06–4.61	1.00
21–30	1/31 (3.2%)	0.61	0.07–5.23	1.00
31–40	2/19 (10.5%)	2.87	0.52–15.98	0.23
41–50	1/24 (4.2%)	0.84	0.10–7.32	1.00
≥51	2/38 (5.3%)	1.14	0.21–6.16	1.00
**Hunting activity**				
Yes	4/27 (14.8%)	6.73	1.41–32.09	**0.02**
No	3/119 (2.5%)			
**Eating armadillo**				
Yes	6/91 (6.6%)	3.81	0.45–32.55	0.26
No	1/55 (1.8%)			
**Frequency of eating**				
Do not eat	1/55 (1.82%)	0.26	0.03–2.24	0.26
1–12 times per year	4/64 (6.25%)	1.76	0.38–8.10	0.70
>12 times per year	2/27 (7.41%)	1.82	0.33–9.95	0.61
**Handling armadillo**				
Yes	6/96 (6.3%)	3.27	0.38–27.93	0.42
No	1/50 (2.0%)			
**Family income**				
<1 minimal wage	5/106 (4.7%)	0.94	0.17–5.06	1.00
1–2 minimal wage	2/40 (5.0%)	1.06	0.20–5.72	

* Leprosy patients previously diagnosed (n = 3) or newly diagnosed (n = 4) during the clinical exam of this study (total patients with leprosy = 7).

** Analysis of risk factors for leprosy infection by the Pearson Chi-square test (χ2) or Fisher’s exact test. OR = Odds ratio. 95% CI = 95% confidence interval. Bold *p* value indicates significance.

### Influence of hunting, cleaning or preparing meat for consumption, or frequency of eating armadillo meat on the anti-PGL-I titer

We collected information from all of the individuals surveyed (n = 146) regarding the level of exposure they had with armadillos based on different behavioral aspects and dietary preferences. This included detailed questions about various activities, including whether or not individuals hunted armadillos in the surrounding forest; whether they were involved in killing the animals and preparing the meat for cooking or consumption; and the frequency with which they consumed armadillo meat, ranging from not at all to those who ate armadillo meat more than once per month. Of all of the individuals surveyed, 27/146 (18.5%) hunted armadillos in the forest, 96/146 (65.8%) either handled or prepared the meat for consumption, and 91/146 (62.3%) ate armadillo meat at least once during the past year, with 27/146 (18.5%) individuals eating them more than once per month. The percentage of individuals that participated in at least one of these activities (hunting, preparing the meat for consumption, or eating the meat) was 96/146 (65.8%). We then examined the PGL-I titers for each group of individuals based on whether or not they participated in these activities or preferences (Table 2).

**Table 2:** Risk factors of behaviors associated with PGL-I titer in surveyed residents living in the municipality of Belterra, Pará.

Risk factors	N (%)	Median titer	p*	PGL-I positive^α^ (%)	OR	95% CI
**Hunting activity**						
Yes	27 (18.5%)	0.38	0.99	18 (66.7%)	1.22	0.50–2.94
No	119 (81.5%)	0.38		74 (62.2%)		
**Handling armadillo**						
Yes	96 (65.8%)	0.38	0.90	60 (62.5%)	0.94	0.46–1.91
No	50 (34.2%)	0.37		32 (64.0%)		
**Eating armadillo**						
Yes	91 (62.3%)	0.38	0.50	58 (63.7%)	1.09	0.54–2.17
No	55 (37.7%)	0.36		34 (61.8%)		
**Frequency of eating armadillo**						
Do not eat	55 (37.7%)	0.36	**0.03**	34 (61.8%)	0.92	0.46–1.84
1–12 times per year	64 (43.8%)	0.36	**0.01**	38 (59.4%)	0.76	0.39–1.49
>12 times per year	27 (18.5%)	0.53	**#**	20 (74.1%)	1.77	0.64–4.89

* PGL-I titer values compared by Mann-Whitney test.

^**α**^ Positive when the PGL-I titer >0.295 O.D. by ELISA. OR = Odds ratio. 95% CI = 95% confidence interval.

# Statistical analysis when compared with the group eating >12 times per year. Bold *p* value indicates significance.

**Fig 6:**
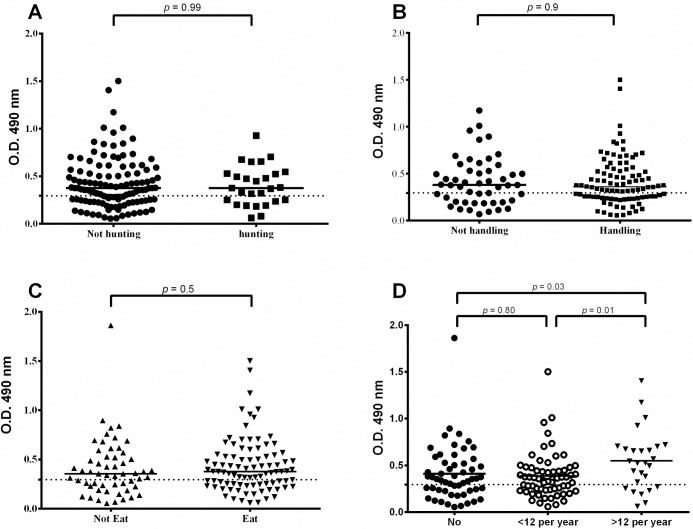
Analysis of anti-PGL-I ELISA titer of 146 individuals based on group behaviors. Behaviors were associated with the type of contact with armadillos, including A) hunting armadillos in the forest, B) handling or preparing armadillo meat for cooking or consumption, C) eating armadillo meat and D) frequency of consuming armadillos in the diet.

Overall, there was no statistical difference in the anti-PGL-I titers of those individuals who hunted or did not hunt armadillos (*p* = 0.99); those who did or did not handle armadillo meat in preparation for consumption (*p* = 0.90); or between individuals who did not eat or ate armadillos (*p* = 0.50) (Fig 6A–6C). However, when we subdivided individuals who ate armadillo meat in low to moderate frequencies (less than once per month) versus those who ate it frequently (more than once per month), there was a significantly higher median anti-PGL-I titer in those individuals who consumed armadillo meat most frequently (*p* = 0.01) with a higher risk (OR = 1.77, 95% CI [0.64–4.89]) (Fig 6D) compared to other groups.

## Discussion

The mammalian order *Xenarthra* includes sloths, anteaters and armadillos. Armadillos are observed only in the Americas, having ten known genera composed of 21 different species in the wild, and are known reservoirs for a number of bacterial and parasitic pathogens, including *Mycobacteria*, *Trypanosoma*, *Toxoplasma*, *Sarcocystis*, *Leptospirosis*, *Sporothrix*, *Chagas and Paracoccidioides* [35,36]. *D*. *novemcinctus*, commonly known as the nine-banded armadillo in the U.S. or chicken-armadillo in Brazil, is the only species whose range includes North, Central and South America [37], and are ground burrowers and opportunistic feeders (almost 500 separate food items, mostly insects, make up their diet). Nine-banded armadillos extended their range from Mexico into Texas some time during the 1800s, and then eventually increased their range north and east into the gulf states of the southern U.S. [38]. In the early 1970s, it was determined that armadillos were capable of sustaining the growth of *M*. *leprae* to extremely high bacillary loads (up to 10^11^ bacilli per gram of tissue), with around 80% of the animals showing some level of susceptibility to experimental infection [39]. Shortly after this, in 1975 it was discovered that wild armadillos living in Texas and Louisiana were naturally infected with *M*. *leprae*, and serological studies of archived armadillo serum samples for anti-PGL-I antibodies indicated that they had likely shown this biomarker of infection as early as the 1960s. Other investigators, using different approaches to identify acid-fast bacilli by histopathology, *M*. *leprae* DNA (such as RLEP sequence) by PCR or serological studies to show anti-PGL-I antibody positivity revealed evidence of infection in armadillo species in a number of countries in Central and South America, including Mexico [40], Colombia [41], and Argentina [42]. In Brazil, several groups have reported the possibility of *M*. *leprae* infection in armadillos in different regions, including in the southeastern state of Espírito Santo [43] and more recently in the northeast in Ceará state [44,45]. Another case control study indicated that human contact with armadillos increased the risk of leprosy in Espírito Santo, Brazil [46]. There was one previous study that examined whether the consumption of armadillo meat had an effect on the incidence of leprosy, but no association was found [47]. However, this study examined individuals living in the southern state of Paraná, considered a medium endemic area (<1 new case per 10,000 population), which is around fifty times lower than in the state of Pará, and there was no evidence presented that armadillos in this area were infected with *M*. *leprae*. In a recent study of 98 marmosets captured from different regions of Brazil to look for Ag85B and RLEP DNA by PCR, none were found to be positive by this method [48], although 14 were found to be positive for *rpoB*, another mycobacterial genetic marker. Nevertheless, the likelihood that environmental reservoirs, including armadillos, amoeba [49,50] and most recently, red squirrels in Scotland and the U.K. [51,52], could play a role in *M*. *leprae* persistence and transmission to humans has been increasingly cited as a real possibility [53], which would necessitate a different approach to leprosy control and prevention.

Although the link between zoonotic *M*. *leprae* infection in armadillos and transmission of these particular unique strain types to humans has been firmly established in the southern U.S., the actual mechanism of transmission of *M*. *leprae* between armadillos and humans would be difficult to determine because of the lack of being able to grow this species *in vitro* in axenic medium. Possible routes of infection that have been proposed include inhalation of particles of soil contaminated by infected armadillos during the process of gardening activity [54] or from contact with contaminated soil or water samples in leprosy endemic areas [55–57]. The evidence for transmission of *M*. *leprae* to humans from these environmental sources is somewhat circumstantial, and has relied mainly on the identification of certain genetic markers, including SNP type and variable nucleotide tandem repeats (VNTR) found in *M*. *leprae* DNA isolated from these sources and matching these with strain types existing in the human population living in the same area. A more definitive transmission link between distinct SNP subtypes circulating in armadillos and those found in human leprosy patients in the southern and southeastern U.S. has been more rigorously confirmed by whole genome sequencing of *M*. *leprae* from tissue or biopsy specimens [58,59], which is currently being pursued with samples from Pará, Brazil. Notably, in this study we used four different techniques to demonstrate the presence of *M*. *leprae* in the armadillo tissue samples. First, PCR was used show RLEP positivity in 62% of the tissues sampled, with confirmation of a match of the RLEP sequence to that already published; second, auramine/rhodamine and SYBR Gold staining showed positively stained bacilli within histological sections, techniques specifically used to identify mycobacteria *in situ*; third, the *M*. *leprae*-specific antigen, PGL-I, was localized *in situ* with a polyclonal rabbit antibody; and finally by showing acid fast staining of bacilli in wild armadillo spleen sections by the Fite Faraco technique.

The total number of new cases of leprosy in the U.S. has remained relatively constant at ~200 per year, while the new case detection rate in 2016 in Brazil of around 25,000 cases in 207 million people translates to 1.2 per 10,000 population. Since armadillos occur at very high numbers in many rural areas in Brazil and the new case detection rate in humans has been considered hyperendemic in the Amazon region for a long time, it is extremely likely that the introduction of *M*. *leprae* in armadillos due to interactions with infected humans is not a recent event, particularly as the percentage of infection in these animals exceeds that found in the southern U.S.

Due to the already high percentage of anti-PGL-I positivity in the general population, we initially wondered whether exposure to armadillos would have a measurable effect on increasing the rates of positivity of this biomarker of infection. However, recording the habits of some of the families, we discovered certain practices that could dramatically influence exposure to *M*. *leprae* from armadillos. Hunters in the villages who capture wild armadillos in the surrounding forest sometimes bring the animals to their home alive where they are kept in an enclosure inside the house for up to six months while being well-fed to increase their body weight and sometimes even bathed like a pet. If an animal was infected and shedding bacilli, this would greatly facilitate infection of individuals living in this dwelling via the aerosol route [60]. Another risk of exposure to viable *M*. *leprae* would likely occur by killing the animal and handling the meat for consumption, as blood or other tissue fluids could gain entry through any cuts in the skin. Cooking the meat would effectively kill *M*. *leprae* bacilli and render infection by eating cooked meat a very low probability. However, another practice in certain areas was for individuals to prepare a kind of raw liver and onion ceviche. Consumption of raw meat, particularly the liver of armadillos, which is one of the main organs where *M*. *leprae* growth is highest, would be considered a very high risk behavior and among the practices most likely leading to successful infection. Although we did not see a significant increase in the anti-PGL-I titer comparing groups of individuals who did or did not eat armadillos, the finding that individuals who consumed armadillo meat frequently (more than once per month and even up to twice per week) had a significantly higher titer indicates that this behavior has an effect on the frequency of infection. Although capturing of wild armadillos for consumption is prohibited in Brazil, it is obvious that a good percentage of people in poor rural areas enjoy and utilize this source of dietary protein, and this preference would be hard to change even by educating them about this potential source of *M*. *leprae* infection. In addition, the WHO has only considered strategies for intervention and treatment of cases involving human-to-human transmission. Interruption of infection by zoonotic transmission (armadillo-to-human) has not been addressed, and would be a difficult challenge for the WHO [61]. Nevertheless, determining the extent of infection in these wild animals and applying whole genome sequencing to identify strain types circulating in armadillos and human populations interacting with them is important to clarify the relative risk that nonhuman reservoirs have in the transmission of this (and perhaps other) tropical diseases and may help to improve strategies to combat leprosy.
